# Genetic diversity and haplotype analysis of yak and sheep echinococcal cysts isolates from the mitochondrial *cox*1 gene in parts of Tibet, China

**DOI:** 10.3389/fvets.2022.1016972

**Published:** 2022-11-07

**Authors:** Shijie Fan, Xialing Zhao, Danqulamu, Bin Shi, Wenqiang Tang, Hailong Dong, Chenyang Xia

**Affiliations:** ^1^College of Animal Sciences, Tibet Agriculture and Animal Husbandry College, Linzhi, China; ^2^Institute of Animai Science of Tibet Academy of Agricultural and Animal Husbandry Sciences, Lhasa, China

**Keywords:** hydatid disease, *Echinococcus granulosus* sensu stricto, yak, sheep, haplotype, China, Tibet

## Abstract

Echinococcosis, also known as hydatid disease, is caused by the metacestode stage of the species cluster *Echinococcus granulosus* sensu lato (*E. granulosus* s.l.). It is almost widespread worldwide, especially in countries and regions dominated by animal husbandry. It is a major parasitic disease that seriously endangers human health, public health safety, environmental safety, and the development of animal husbandry production in western China. In this study, the mitochondrial *cox*1 gene was used to analyze the genetic diversity and haplotype of bovine and sheep echinococcal cysts isolated in Tibet. *Echinococcus granulosus* sensu stricto (*E. granulosus* s.s., G1, G3) was still the dominant species in the infected samples of yak and sheep in some parts of Tibet. Through haplotype analysis, Hap_1 was deemed the dominant haplotype, 14 of the 20 haplotypes were similar to the reference sequence previously published in Genbank, and the rest of the 6 haplotypes were found for the first time. Through Tajima's D value, neutral test Fu's Fs analysis, and haplotype network map, it can be concluded that *Echinococcus* population expansion has occurred in Xigaze, Tibet. This study provides basic data for understanding the genetic characteristics, epidemiology, and control of echinococcosis in this area.

## Background

Echinococcosis, also known as hydatid disease, is caused by the metacestode stage of the species cluster *Echinococcus granulosus* sensu stricto (*E. granulosus* s.s.). It is parasitic in the liver, lungs, and other organs of humans and animals, mainly causing mechanical compression, toxins, and allergic reactions at parasitic sites (1). Echinococcosis is an almost widespread disease worldwide, especially in rural settings in most parts of the world, such as the Middle East, Mediterranean Europe, and Central Asia (2). Khan et al. (3) conducted an epidemiological survey on slaughterhouses in two regions of Pakistan in 2017 and found that the prevalence of diseased animals in cattle was 27%. Mohaghegh et al. (4) reported that the infection rate of echinococcosis in sheep was 5.1 and 74.4% in Iranian ruminants. Echinococcosis is also prevalent in western China. In China, seven provinces and autonomous regions, including Xinjiang, Qinghai, Gansu, Ningxia, Inner Mongolia, Tibet, and Sichuan, are seriously endemic areas of echinococcosis. The analysis of published data showed that the comprehensive prevalence rate of echinococcosis in sheep in China was 30.9% from 1983 to 2020 (5), and the comprehensive prevalence rate of echinococcosis in cattle in China was 17.3% from 2000 to 2020 (6). It is a major parasitosis that seriously endangers human health, public health safety, environmental safety, and the production and development of animal husbandry in western China (7).

In the past few decades, scientists have studied the genetic diversity of *Echinococcus* according to the infection of various intermediate hosts based on the mitochondrial DNA of *Echinococcus* (8). It has been shown that *Echinococcus* contains 10 genotypes (G1–G10), which have been identified as different species, including *E. granulosus* s.s. (G1 and G3). Recently, Kinkar et al. (9) represented that *E. granulosus* s.s. is composed of G1 and G3 genotypes, while G2 is considered a microvariant of G3, *Echinococcus equinus* (G4), *Echinococcus ortleppi* (G5), *Echinococcus canadensis* (G6–G10), and *Echinococcus felidis* (lion strain) (10). The G1 genotype is still the most widely distributed genotype in China and even in the world. Bart et al. (11) used mitochondrial cytochrome oxidase subunit 1 (*cox*1) gene sequencing to discover the existence of G1 and G6 genotypes of *Echinococcus* in Xinjiang, China. Yang et al. (12) used atp6 gene sequencing to verify that the genotype of *Echinococcus* in southwestern Sichuan is still dominated by G1. Yantao Wu et al. (13) found *Echinococcus* G10 genotypes in the Qinghai-Tibet Plateau by *cox*1 gene sequencing, and the results of NADH dehydrogenase subunit 1 (*nad*1) gene sequencing also confirmed this discovery. In addition, G3, G7, and G9 genotypes were sporadically distributed in China (14).

Mitochondrial genes evolve faster than nuclear rRNA genes; they are advantageous for discriminating against closely related species and population genetic studies (15). Regarding intraspecific variation, the *cox*1 gene sequence of *E. granulosus* s.s., haplotypes G1 and G3, possess relatively higher genetic variation than the *nad*1 gene sequences (16). Therefore, this study considered using only the *cox*1 gene as the genetic marker to identify the echinococcal cysts of yak and sheep in Xigaze City, Tibet Autonomous Region of China, and to determine the haplotype.

## Materials and methods

### Study area

**Figure 1:**
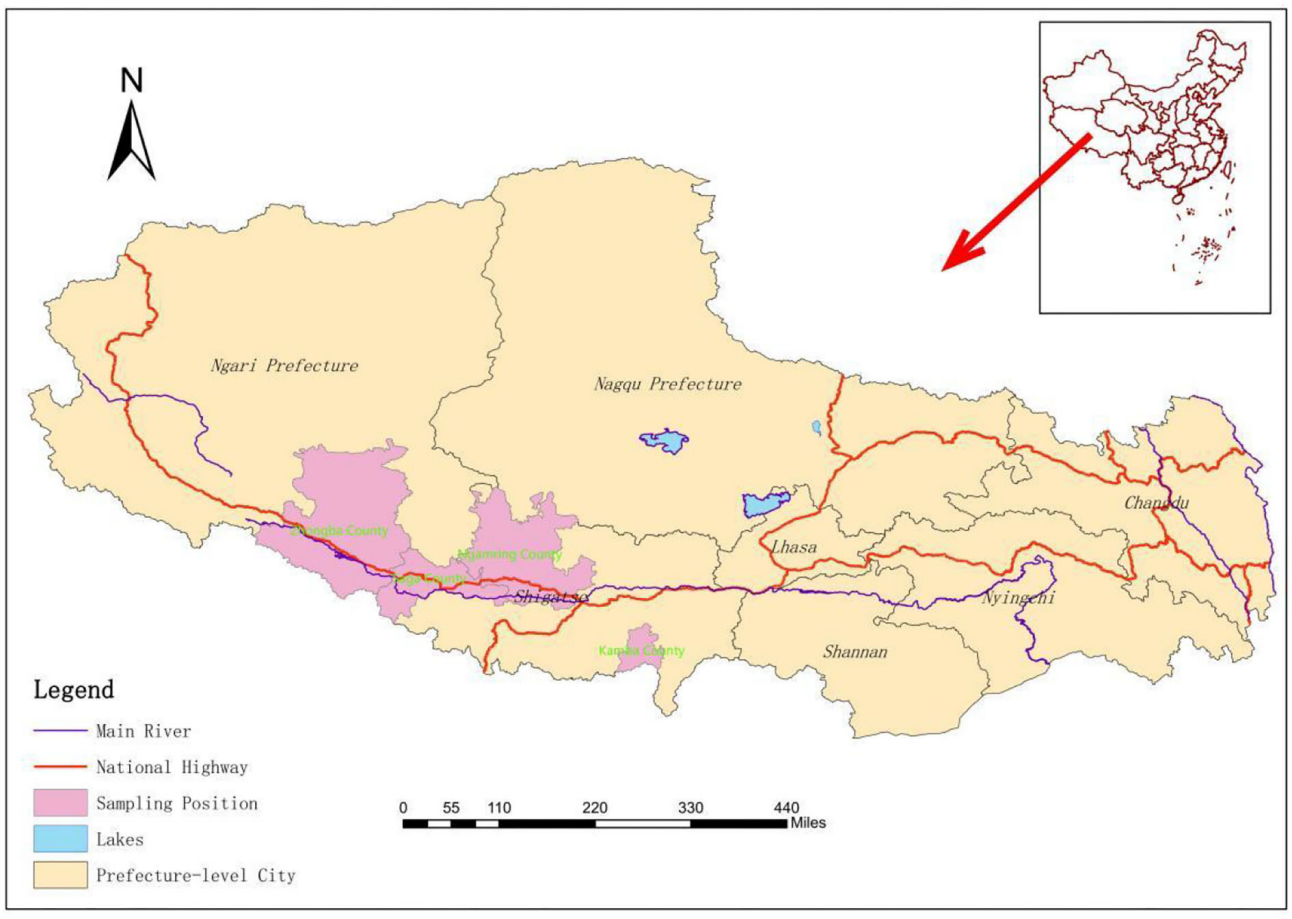
Geographic origin of the livestock detected in the study.

This study mainly focuses on Saga County, Ngamring County, Kamba County, and Zhongba County (Figure 1) in Xigaze City, Tibet Autonomous Region, which is located on the southwest border of the People's Republic of China, southwest of the Qinghai-Tibet Plateau, and shares borders with Nepal, Bhutan, India, and other countries. The average altitude is more than 4,000 m, and the climate is cool and cold, which belongs to the temperate semiarid climate of the plateau. The study area is mainly animal husbandry and semi-agricultural semi-animal husbandry (17).

### Collection of samples

Echinococcal cysts (*n* = 132) were collected during the slaughter season in December 2017 and December 2021 from *Bos grunniens* (yak) and *ovis* (sheep and goats) in four counties of Xigaze City. In 2017, 50 samples were collected and the rest were collected in 2021. Echinococcal cysts were found in the liver and the lung. The cysts were numbered and stored in ziplock bags at −80°C.

The germinal layer and contents of the cyst and contents of calcified sacs without stratum corneum and germinal layer were selected, and the tissue samples were washed with phosphate-buffered saline (PBS) three times. The samples of the germinal layer of 30 mg were chopped and treated with a certain amount of ultrapure water for cell suspension.

### DNA extraction and PCR amplification of gene fragments

The chopped germinal layer was completely broken using a fully automatic sample rapid grinding instrument (Tlssuelyser-24, Shanghai Jingxin Technology, China), and then the genomic DNA was extracted using the centrifugal column type of blood (catalog number: DP304, Tiangen, China), cell and tissue genomic DNA extraction kit.

According to the *cox*1 gene sequence (Genbank accession number: AF297617) in the gene bank and referring to the primer design of Guo et al. (18), PCR primers, including forward (5′-GTTTAGGGGCTGGTGTTGGT-3′) and reverse (5′-CCGTCTTCACATCCAACCCA-3′) primers, were used to amplify part (546 bp) of the *cox*1 gene.

The PCR reaction comprised the following (50 μl): 25 μl of 2 × Taq PCR Master Mix (Tsingke Biotechnology Co., Ltd., China), 2 μl of upstream and downstream primers, 4 μl of template, and 17 μl of ddH_2_O completion were mixed and centrifuged slightly.

The amplification conditions were as follows: 3 min of pre-denaturation at 95°C, 15 s of denaturation at 95°C, 15 s of annealing at 55°C, and 35 s of extension at 72°C, for a total of 30 cycles, and finally 5 min of extension at 72°C. At the same time, ddH_2_O was used instead of template DNA as blank control, and ordinary PCR was used for PCR amplification (Biometra TOne 96G, Germany). PCR amplicons were checked on 2.0% agarose gels and visualized under ultraviolet light. Subsequently, the positive identification results were recorded and photographed, and the positive PCR products were sent to Tsingke Biotechnology Co., Ltd. for two-way sequencing.

### Haplotype analysis and phylogenetic analysis

After sequencing, the sequences of the *cox*1 gene obtained by sequencing were spliced using the MegAlign Pro program in the DNAstar software, and the genotype was determined using BLAST functional comparison on the NCBI website (19). Combined with the alignment results, the *cox*1 gene sequence was aligned with the alignment sequence using the MEGA7 software. According to the results of Fasta format alignment, the haplotype of the *cox*1 gene was analyzed using the DnaSP5 software, and the diversity of haplotype was analyzed by population index and neutral index (1). The haplotype network map of all haplotypes of the *cox*1 gene was constructed using the popart software. The *cox*1 gene sequences of several species of *Echinococcus* were downloaded from GenBank. *Taenia multiceps* (GenBank accession number: NC_012894) was used as the outgroup in phylogenetic analysis. The neighbor-joining (NJ) tree was inferred using the MEGA 7 software. Nodal support was assessed by bootstrapping with 1,000 replicates (20).

## Results

### Results of gene sequencing and sequence alignment

**Figure 2:**
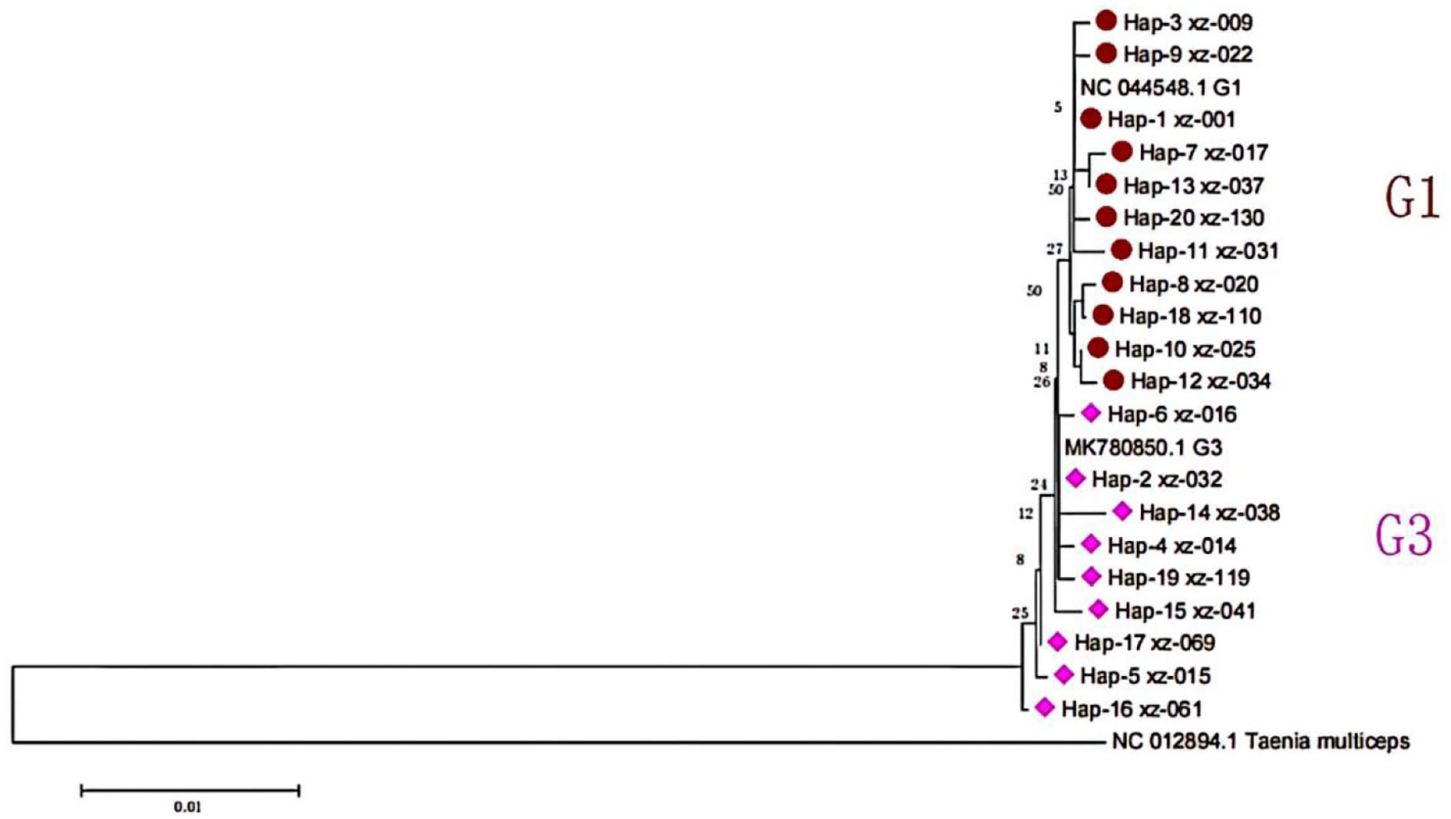
The phylogeny of *E. granulosus* s.l. based on *cox*1 mitochondrial sequences.

The use of anthelmintics in more calcified sacs has increased dramatically year by year, especially in the early stages of the growth of *Echinococcus granulosus*. DNA extraction from the calcified sacs is difficult. Therefore, 105 gene sequences were obtained from 132 cattle and sheep samples. Among them, 51 strains were from yak and 54 from sheep. According to BLAST alignment and phylogenetic tree analysis (Figure 2), the identified haplotypes in 2 distinct clades were positioned along the reference sequences. All sequences were identified as *E. granulosus* s.s., 80 sequences of 11 haplotypes were identified as G1 genotypes, and 25 sequences of 9 haplotypes were identified as G3 genotypes. The relevant information representing the nucleotide sequence of the *cox*1 gene of *E. granulosus* has been successfully uploaded to GenBank (accession number is OP278730 to OP278833).

By comparing with the reference sequence NC044548, 10 polymorphic sites were identified in the G1 gene sequence, accounting for ~2.27% of the total number of analyzed sites, and the differences within G1 were 0–0.45%. Among them, there were 8 parsimony information sites. In addition, the base frequencies of the gene sequences were not equal, and the average contents of A, T, G, and C bases were 15.68, 51.36, 21.37, and 11.59%, respectively. Among them, the content of A+T was 67.04%, and G+C was 32.96%. Meanwhile, there were only transformations in all mutations in the nucleotide sequence, and there were no transversions, deletions, and insertions. Compared with the reference sequence MK780850.1, in the G3 gene sequence, 430 conserved sites and 10 polymorphic sites were identified. The differences within G3 were 0% to 0.68%, and the *cox*1 sequence differences between the G3 and G1 genotypes were 0.22% to 0.90% in this study. In the G3 gene sequences, there were 5 single variation polymorphic sites. In addition, the base frequencies of the gene sequences were not equal, and the average contents of A, T, G, and C bases were 15.64, 51.45, 21.40, and 11.51%, respectively. Among them, the content of A+T was 67.09%, and G+C was 32.91%.

### Haplotype characteristics of the *cox*1 gene

**Figure 3:**
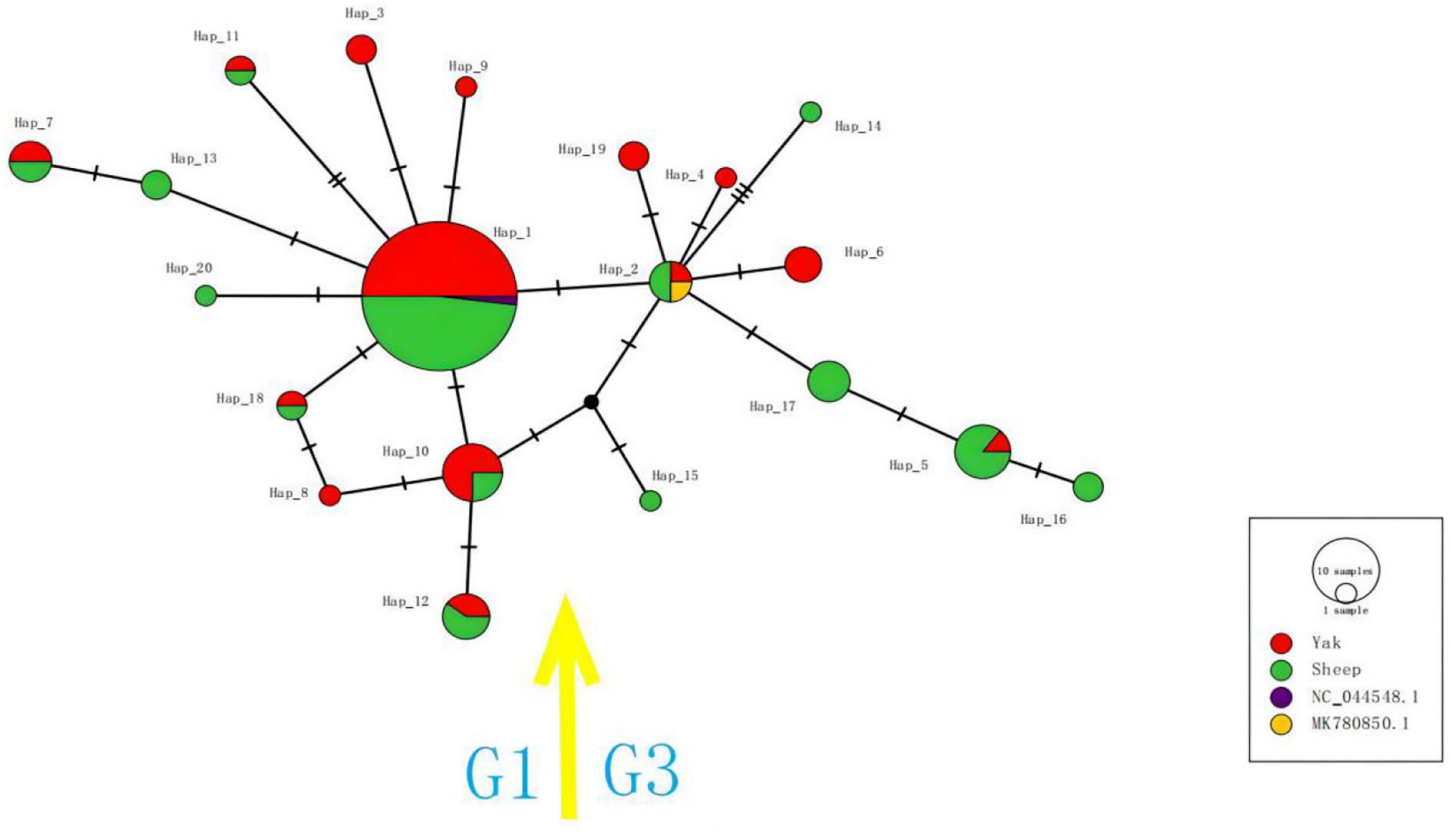
The haplotype network for the *cox*1 gene of *E. granulosus* s.s. Circle sizes are proportional to the frequency of each haplotype. The number of mutations separating haplotypes is indicated by dash marks. Hap: haplotype.

In this study, the *cox*1 gene sequences of 105 samples successfully amplified by PCR could be classified into 20 haplotypes, which were named Hap_1 to Hap_20 (Figure 3). Hap_2, Hap_4, Hap_5, Hap_6, Hap_14, Hap_15, Hap_16, Hap_17, and Hap_19 haplotypes were all of the G3 genotypes, and other haplotypes were all of the G1. The predominant haplotype of G1 was Hap_1 in this study, accounting for 67.5% (54/80). While there was no dominant haplotype in the G3 genotype, the 14 haplotypes detected in this study were 100% similar to the reference sequence previously published in Genbank. The other 6 haplotypes that have not appeared in any references (blue in Table 1) were the first discovered in this study. Hap_3, Hap_4, Hap_6, Hap_8, Hap_9, and Hap_11 haplotypes were only found in Saga County; Hap_14 and Hap_15 haplotypes were only found in Kamba County; and Hap_16, Hap_17, Hap_18, Hap_19, and Hap_20 haplotypes were only found in Zhongba County, indicating that the strains in the same area have a similar genetic background. Hap_13 was found in Kamba County and Zhongba County; Hap_2 and Hap_5 were found in Kamba County and Saga County; Hap_7, Hap_10, Hap_12, and Hap_13 were found in Zhongba County and Saga County, indicating that *E. granulosus* has a similar genetic background in this area (21).

**Table 1:** Haplotype assignment and sample distribution of *cox*1 gene sequence.

**Serial number**	**Haplotype name**	**Number of isolates**	**Genotype and host (sheep, yak)**
1	Hap_1	53	xz-001 (Yak); xz-002 (Yak); xz-003 (Yak); xz-006 (Yak); xz-007 (Yak); xz-008 (Yak); xz-012 (Yak); xz-013 (Yak); xz-019 (Yak); xz-023 (Sheep); xz-024 (Yak); xz-026 (Yak); xz-027 (Yak); xz-028 (Yak); xz-035 (Yak); xz-042 (Yak); xz-050 (Sheep); xz-051 (Yak); xz-053 (Sheep); xz-056 (Sheep); xz-058 (Sheep); xz-062 (Sheep); xz-063 (Sheep); xz-064 (Yak); xz-065 (Sheep); xz-066 (Sheep); xz-067 (Yak); xz-071 (Yak); xz-072 (Yak); xz-073 (Yak); xz-074 (Sheep); xz-077 (Yak); xz-079 (Yak); xz-081 (Yak); xz-082 (Yak); xz-086 (Sheep); xz-089 (Sheep); xz-090 (Sheep); xz-099 (Sheep); xz-104 (Sheep); xz-108 (Sheep); xz-111 (Sheep); xz-113 (Yak); xz-114 (Yak); xz-115 (Sheep); xz-116 (Sheep); xz-117 (Sheep); xz-118 (Sheep); xz-121 (Sheep); xz-124 (Sheep); xz-126 (Sheep); xz-129 (Sheep); xz-131 (Sheep)
2	Hap_2	3	xz-032 (Sheep); xz-043 (Sheep); xz-054 (Yak)
3	Hap_3	2	xz-009 (Yak); xz-029 (Yak)
4	Hap_4	1	xz-014 (Yak)
5	Hap_5	7	xz-015 (Yak); xz-055 (Sheep); xz-068 (Sheep); xz-087 (Sheep); xz-088 (Sheep); xz-091 (Sheep); xz-092 (Sheep)
6	Hap_6	3	xz-016 (Yak); xz-036 (Yak); xz-083 (Yak)
7	Hap_7	4	xz-017 (Yak); xz-018 (Yak); xz-057 (Sheep); xz-112 (Sheep)
8	Hap_8	1	xz-020 (Yak)
9	Hap_9	1	xz-022 (Yak)
10	Hap_10	8	xz-025 (Yak); xz-030 (Yak); xz-033 (Yak); xz-039 (Yak); xz-075 (Sheep); xz-078 (Yak); xz-080 (Yak); xz-128 (Sheep)
11	Hap_11	2	xz-031 (Yak); xz-095 (Sheep)
12	Hap_12	5	xz-034 (Yak); xz-052 (Sheep); xz-059 (Sheep); xz-084 (Yak); xz-096 (Sheep)
13	Hap_13	2	xz-037 (Sheep); xz-102 (Sheep)
14	Hap_14	1	xz-038 (Sheep)
15	Hap_15	1	xz-041 (Sheep)
16	Hap_16	2	xz-061 (Sheep); xz-103 (Sheep)
17	Hap_17	4	xz-069 (Sheep); xz-100 (Sheep); xz-012 (Sheep); xz-123 (Sheep)
18	Hap_18	2	xz-110 (Sheep); xz-132 (Yak)
19	Hap_19	2	xz-119 (Yak); xz-120 (Yak)
20	Hap_20	1	xz-130 (Sheep)

The haplotype network map of G1 uses Hap_1 as the network center, and Hap_1 was the largest haplotype population of this genotype. Other haplotypes were linked to it by 1–2 steps of the gene mutation process. Several haplotypes of this genotype were host specific. Among them, Hap_3, Hap_8, and Hap_9 haplotypes originated in the yak, while Hap_13 and Hap_20 all originated in sheep. The haplotype network map of the G3 gene showed that Hap_2 was a network-centered haplotype, and other haplotypes were linked to it by 1–3 steps of gene mutation. Among them, Hap_4, Hap_6, and Hap_19 haplotypes derived from yak strains have a similar genetic distance to Hap_2 haplotypes, indicating that their genetic correlation was strong, while the structure of Hap_15 and Hap_16 haplotypes was relatively complex.

### Analysis of nucleotide diversity neutralization index

According to the standard proposed by Grant et al. (22), the critical value of haplotype diversity was 0.5 and that of nucleotide diversity was 0.005. The higher the value of haplotype and nucleotide diversities, the higher the diversity of the population. Therefore, the haplotype diversity of the G1 gene was higher and the nucleotide diversity was lower (Hd = 0.555 ± 0.064, Pi = 0.00205 ± 0.00031). Tajima's D value was −1.45530, which was non-significantly negative. The neutral test Fu's Fs was −0.57571, which was non-significantly negative (Table 2). The haplotype diversity and nucleotide diversity of the G3 gene were higher (0.873 ± 0.039, Pi = 0.00456 ± 0.00606). Tajima's D value was −0.81701, which was non-significantly negative. The neutral test Fu's Fs was −1.21867, which was non-significantly negative.

**Table 2:** Diversity and neutrality indices obtained using nucleotide data of the *E. granulosus* s.s. *cox*1 gene.

**Genotype**	**Number of isolates**	**Number of haplotypes**	**Haplotype diversity (Hd)**	**Nucleotide diversity (Pi)**	**Tajima's D**	**Statistical significant**	**Fu's Fs**
G1	80	11	0.555 ± 0.064	0.00205 ± 0.00031	−1.45530	*P* > 0.1	−0.57571
G3	25	9	0.873 ± 0.039	0.00456 ± 0.00606	−0.81701	*P* > 0.1	−1.21867

## Discussion

Echinococcosis causes huge economic losses and serious public health risks worldwide, and more than 7 million livestock are affected every year (23). In China, the harm of echinococcosis cannot be ignored. As one of the most popular areas in China, the average infection rate of livestock in the Tibet Autonomous Region has reached 11.84% (24). It is particularly important to analyze the gene sequence of *Echinococcus* in order to judge the genotype and genetic diversity. In this study, all sequences were identified as *E. granulosus* s.s. (G1, G3), confirming that the G1 genotype of *E. granulosus* was still the most popular genotype in Tibet (25). There were 20 haplotypes of the *cox*1 gene in this study, and 14 haplotypes were 100% similar to the reference sequence previously published in Genbank. Among them, Hap_1 was the most common haplotype of the G1 genotype, and the sequence of Hap_1 was the same as that of bovine isolates reported in Pakistan (6). The other 100% similar haplotypes were the same as the cattle and sheep sources reported in Algeria (26), the human sources reported in Kyrgyzstan (27), and the human sources in Qinghai Province (28) and Sichuan Province (29). Sheep were first domesticated in the Middle East around 12,000 BC and then spread to Europe, Africa, America, and Asia. Recent studies have shown that sheep parasites are also spreading, which has led to an increase in parasite populations in these areas (30). The “Silk Road” between China and the Middle East also accelerated the spread of the parasite through livestock trade (31). The author analyzes the causes of gene flow in the following aspects: first, the migration of final hosts such as stray dogs, wolves, and foxes is the main reason for gene flow; second, the trade of yak and sheep resulting in gene flow and migration, with individual gene sites converting over time in the local environment.

We found that the haplotype and nucleotide diversities of the G3 gene were higher in the study area, while the haplotype diversity of the G1 gene was higher and the nucleotide diversity was lower. This was similar to the results of John Asekhaen Ohiolei et al. (32) on the mtDNA of yak and sheep *Echinococcus* in Lhasa and Xigaze. *Echinococcus* genes flow across multiple hosts in a certain region, which increases the chances of nucleotide and haplotype diversity. After yak and sheep were slaughtered, the fresh offal with cysts was discarded to canines, and then the livestock life cycle was completed. At the same time, the life cycle of wild animals was completed in the same living environment, and all the intermediate hosts are vulnerable to definitive hosts in the same areas. The reason for the difference between the G1 and G3 genotypes was that there was a great difference in sample size between them, which was also the deficiency in data processing in this study. There are a large number of domestic and wild intermediate hosts in the Qinghai-Tibet Plateau, and echinococcosis is serious in some areas. In order to understand more about the genetics of echinococcus, not only yak and sheep but also other vulnerable wild animals should be studied.

In this study, the maximum differences between G1 and G3 gene sequences were 0.9%, which was far higher than the 0.6% obtained by Bowles et al. (33). Many studies show that mitochondrial gene variation is much higher than reported by Gulay Vural et al. (34). Based on the haplotype and genetic distance analyses, the differences in *cox*1 sequences between G3 and G1 genotypes were very similar. Genotypes G1–G3 cluster firmly together to form the taxon *E. granulosus* s.s., which is acknowledged by many researchers (35).

Tajima's D is nonsignificantly negative, and the neutral test Fu's Fs was nonsignificantly negative. Under the same conditions, Fu's Fs test is sensitive to the recent expansion of the population (36). Combined with the haplotype network map, it can be considered that Echinococcus population expansion has occurred in Xigaze, Tibet. This inference phenomenon has the same characteristics as the results of the study of the mtDNA of *Echinococcus* in southwest China (37).

## Conclusion

*Echinococcus granulosus* s.s. (G1, G3) was still the dominant species in the infected samples of yak and sheep in some parts of Tibet. Through haplotype analysis, it was found that Hap_1 was the most common among the 20 haplotypes, 14 haplotypes were similar to the reference sequence previously published in Genbank, and the remaining 6 haplotypes were found for the first time. Through Tajima's D value, neutral test Fu's Fs analysis, and haplotype network map, it can be considered that *Echinococcus* population expansion has occurred in Xigaze, Tibet.

## Data availability statement

The original contributions presented in the study are included in the article/Supplementary files, further inquiries can be directed to the corresponding author.

## Ethics statement

The animal study was reviewed and approved by Institute of Animai Science of Tibet Academy of Agricultural and Animal Husbandry Sciences.

## Author contributions

CX, HD, and SF conceived, designed, and coordinated the study. XZ, D, BS, and WT commented on revisions and improvements to this study. SF did experiments and gene sequence analysis. All authors contributed to the data analysis and the preparation of the manuscript. All authors read and approved the final manuscript.

## Funding

Funding was provided by the Research and Integrated Demonstration of Prevention and Control Technology of Major Diseases in Sheep. Award number: XZ202101ZD0001N.

## Conflict of interest

The authors declare that the research was conducted in the absence of any commercial or financial relationships that could be construed as a potential conflict of interest.

## Publisher's note

All claims expressed in this article are solely those of the authors and do not necessarily represent those of their affiliated organizations, or those of the publisher, the editors and the reviewers. Any product that may be evaluated in this article, or claim that may be made by its manufacturer, is not guaranteed or endorsed by the publisher.
