# Supplementary material for: Genetic diversity and haplotype analysis of yak and sheep echinococcal cysts isolates from the mitochondrial cox1 gene in parts of Tibet, China
Source: Front Vet Sci. 2022 Nov 7;9:1016972. doi: 10.3389/fvets.2022.1016972 (PMC9678366; doi:10.3389/fvets.2022.1016972)
Supplement: Supplementary file 1 [file Table_1.DOCX]

Nucleotide variation positions of the cox1 gene among 20 analyzed haplotypes. A: Adenine, T: Thymine, C: Cytosine, G: Guanine

| Nucleotide  Positions | 17 | 101 | 102 | 107 | 137 | 139 | 143 | 146 | 187 | 230 | 280 | 290 | 314 | 347 | 365 | 380 | 386 | 389 | 392 | 407 |
| --- | --- | --- | --- | --- | --- | --- | --- | --- | --- | --- | --- | --- | --- | --- | --- | --- | --- | --- | --- | --- |
| NC_044548 G1  (Reference  sequence) | T | T | C | T | T | C | T | A | T | C | C | T | C | A | T | T | T | T | T | C |
| Hap_1 |  |  |  |  |  |  |  |  |  |  |  |  |  |  |  |  |  |  |  |  |
| Hap_3 |  |  |  |  | C |  |  |  |  |  |  |  |  |  |  |  |  |  |  |  |
| Hap_7 |  |  |  |  |  | T |  |  |  |  |  |  |  |  | C |  |  |  |  |  |
| Hap_8 |  | C |  |  |  |  |  |  |  |  |  |  | T |  |  |  |  |  |  |  |
| Hap_9 |  |  |  |  |  |  |  |  |  |  | T |  |  |  |  |  |  |  |  |  |
| Hap_10 |  |  |  |  |  |  |  |  |  |  |  |  | T |  |  |  |  |  |  |  |
| Hap_11 |  |  | T |  |  |  |  |  |  |  |  |  |  | G |  |  |  |  |  |  |
| Hap_12 | C |  |  |  |  |  |  |  |  |  |  |  | T |  |  |  |  |  |  |  |
| Hap_13 |  |  |  |  |  |  |  |  |  |  |  |  |  |  | C |  |  |  |  |  |
| Hap_18 |  | C |  |  |  |  |  |  |  |  |  |  |  |  |  |  |  |  |  |  |
| Hap_20 |  |  |  |  |  |  |  |  |  | T |  |  |  |  |  |  |  |  |  |  |
| MK780850.1 G3  (Reference  sequence) | T | T | C | T | T | C | T | A | T | C | C | T | C | A | T | T | T | T | T | T |
| Hap_2 |  |  |  |  |  |  |  |  |  |  |  |  |  |  |  |  |  |  |  |  |
| Hap_4 |  |  |  |  |  |  | C |  |  |  |  |  |  |  |  |  |  |  |  |  |
| Hap_5 |  |  |  | C |  |  |  |  |  |  |  | C |  |  |  |  |  |  |  |  |
| Hap_6 |  |  |  |  |  |  |  |  |  |  |  |  |  |  |  |  | C |  |  |  |
| Hap_14 |  |  |  |  |  |  |  |  |  |  |  |  |  |  |  | C |  | C | C |  |
| Hap_15 |  |  |  |  |  |  |  |  | C |  |  |  | T |  |  |  |  |  |  |  |
| Hap_16 |  |  |  | C |  |  |  |  |  |  |  | C | T |  |  |  |  |  |  |  |
| Hap_17 |  |  |  |  |  |  |  |  |  |  |  | C |  |  |  |  |  |  |  |  |
| Hap_19 |  |  |  |  |  |  |  | G |  |  |  |  |  |  |  |  |  |  |  |  |
